# HuR ablation destabilizes *Foxp3* mRNA and impairs regulatory T cell function, contributing to an autoimmune phenotype

**DOI:** 10.3389/fimmu.2025.1618677

**Published:** 2025-09-26

**Authors:** Fatemeh Fattahi, Jason S. Ellis, Laura Vallance, Kristin Bahleda, Julia Holden, Sarah Socha, Joshua Meier, Francisco Gomez-Rivera, Ulus Atasoy

**Affiliations:** ^1^ Division of Allergy and Clinical Immunology, Department of Internal Medicine, University of Michigan Medical School, Ann Arbor, MI, United States; ^2^ Whitehead Institute for Biomedical Research, Cambridge, MA, United States; ^3^ Division of Allergy-Immunology, Ann Arbor VA Health System, Ann Arbor, MI, United States

**Keywords:** regulatory T cells (Tregs), HuR (ELAVL1), Foxp3 stability, systemic autoimmunity, IPEX syndrome, RNA-binding proteins (RBPs), post-transcriptional regulation, RORγt (Rorc)

## Abstract

**Introduction:**

The RNA-binding protein HuR (Elavl1), a key post-transcriptional regulator, plays a critical role in T cell activation and function by stabilizing target mRNAs. To investigate the role of HuR in regulatory T cell (Treg) function, we generated the Foxp3YFP/Cre HuRfl/fl mouse model.

**Methods:**

In this model, homozygous females and hemizygous males for Foxp3 developed a scurfy-like phenotype displaying autoimmune features, including failure to thrive, splenomegaly, hair loss, tail stippling, and widespread multi-organ immune cell infiltration. Molecular analysis included direct interaction studies between HuR and Foxp3 mRNA to assess mRNA stability, RNA sequencing of YFP⁺ Tregs, Protein-Protein Interaction (PPI) analysis, qPCR, and Treg functional assays.

**Results:**

To our knowledge, this is the first study demonstrating that HuR directly binds and stabilizes Foxp3 mRNA in Tregs, using a novel Treg-specific HuR-deficient mouse model, with implications for autoimmune regulation. Foxp3 mRNA stability and expression were significantly reduced in Tregs from these HuR KO mice, despite higher frequencies of YFP⁺ Tregs. RNA sequencing revealed significant dysregulation of several pathways, including the T helper differentiation pathway, in which Foxp3 played a central role. PPI analysis showed a direct link between Foxp3 and Rorc (encoding RORγt), connecting Foxp3 to the T cell differentiation pathway via IL-23R. Our qPCR analysis supported these findings. Functional assays demonstrated a reduction in the suppressive capacity of HuR-deficient Tregs.

**Conclusion:**

These findings together suggest that ablation of HuR in Tregs disrupts Foxp3 expression and Treg function, likely through dysregulation of T cell differentiation pathways involving RORγt. This potentially contributes to a disrupted Treg–Th17 axis and autoimmune dysfunction. These data suggest that HuR-mediated post-transcriptional regulation contributes to maintaining Foxp3 expression and immune homeostasis, although compensatory mechanisms such as increased IL-10 expression may also be involved.

## 1 Introduction

Regulatory T cells (Tregs) are essential for suppressing excessive immune responses and maintaining immune tolerance. In fact, these specialized CD4^+^ T cells prevent autoimmune diseases by suppressing autoreactive T cells and controlling inflammatory responses (1). The forkhead box P3 (*Foxp3*), a well-known transcription factor that defines Treg identity and function (2–5), specifically expressed in CD4^+^CD25^+^ Tregs and plays a central role in their development, stability, and suppressive function, making it the defining marker of Tregs (2). Foxp3 is critical for preventing excessive immune activation by enabling Tregs to maintain immune homeostasis and self-tolerance (6–8). Mutations or dysregulation of Foxp3 impair Treg function and contribute to the pathogenesis of autoimmune diseases such as systemic lupus erythematosus (SLE), rheumatoid arthritis (RA), and multiple sclerosis (MS), where the immune system attacks self-antigens (9, 10). Notably, the loss of functional *Foxp3* (11–13) or imbalance of its isoforms (14, 15) leads to severe systemic autoimmunity known as immune dysregulation, polyendocrinopathy, enteropathy, X-linked (IPEX) syndrome in humans and the scurfy phenotype in mice, both characterized by catastrophic immune dysregulation and widespread autoimmunity (16, 17). IPEX, which primarily affects males, with female carriers exhibiting mild symptoms, presents early in life with clinical features such as autoimmune thyroid disease, diabetes, eczema, hemolytic anemia, and failure to thrive, often resulting in a poor prognosis due to severe autoimmune manifestations (11–13, 18, 19).

The stability and expression of many mRNAs are tightly regulated by post-transcriptional mechanisms, including the actions of RNA-binding proteins, microRNAs, and signaling pathways that influence mRNA decay, translation, and degradation (10, 20, 21). Among these RNA-binding proteins, HuR (encoded by *Elavl1*) plays a key role by stabilizing target mRNAs, such as those in T cells, through binding AU-rich elements (AREs) within the 3′ untranslated region (UTR), thereby enhancing their stability and translation (20, 22). In contrast, other RNA-binding proteins such as the ZFP36 family (ZFP36, ZFP36L1, ZFP36L2) regulate Treg stability indirectly by limiting excessive cytokine signaling, controlling CTLA-4 recycling, and supporting IL-2/IL-7 responsiveness, maintaining immune homeostasis (23), underscoring the importance of post-transcriptional regulation in maintaining Treg stability.

HuR has been extensively studied in T cell activation by our group and others (22, 24–28), including its role in promoting Th17 differentiation by stabilizing critical transcripts such as RORγt, IRF4, and Runx1, which are required for Th17 lineage commitment and autoimmune responses (29–31). Beyond T cells, HuR has been implicated in broader immunoregulatory and disease-associated functions, including modulation of cytokine mRNAs, Th2 differentiation, and inflammatory responses across multiple immune cell types (26, 28). Furthermore, HuR supports oncogenic mRNA networks and profibrotic pathways in cancer, cardiac, and renal fibrosis, and has been targeted therapeutically by small-molecule HuR inhibitors such as KH-3 (32–37). However, to date, no study has directly examined the role of HuR in regulating Foxp3 expression and Treg function.

To address this knowledge gap, we generated a Foxp3*^YFP/Cre^* HuR*^fl/fl^* mouse in which HuR is selectively deleted in CD4^+^Foxp3^+^ cells, a lineage essential for self-tolerance and immune homeostasis. Treg-mediated immune tolerance is critical for suppressing autoimmunity, as shown by studies demonstrating that restoration of Foxp3 in Treg-deficient mice can reverse systemic inflammation (28). However, the molecular mechanisms that maintain Foxp3 expression and Treg stability remain incompletely understood.

Here, we show that Foxp3^+^ Treg-specific HuR deletion in this model results in abnormal phenotypic features similar to those seen in the scurfy mouse, which lacks functional Tregs due to a Foxp3 missense mutation (16, 38). Our data demonstrate that HuR deficiency leads to reduced Foxp3 expression and stability, partial impairment of Treg function, and disruption of key genes involved in the Treg-Th17 balance, including *Rorc* and *Il23r*. This imbalance between Tregs and Th17 cells, which share common precursors, has been suggested to contribute to autoimmune features (38), consistent with our mouse model findings.

## 2 Materials and methods

### 2.1 Generation of Foxp3*^YFP/Cre^* HuR*^fl/fl^*

**Figure 1:**
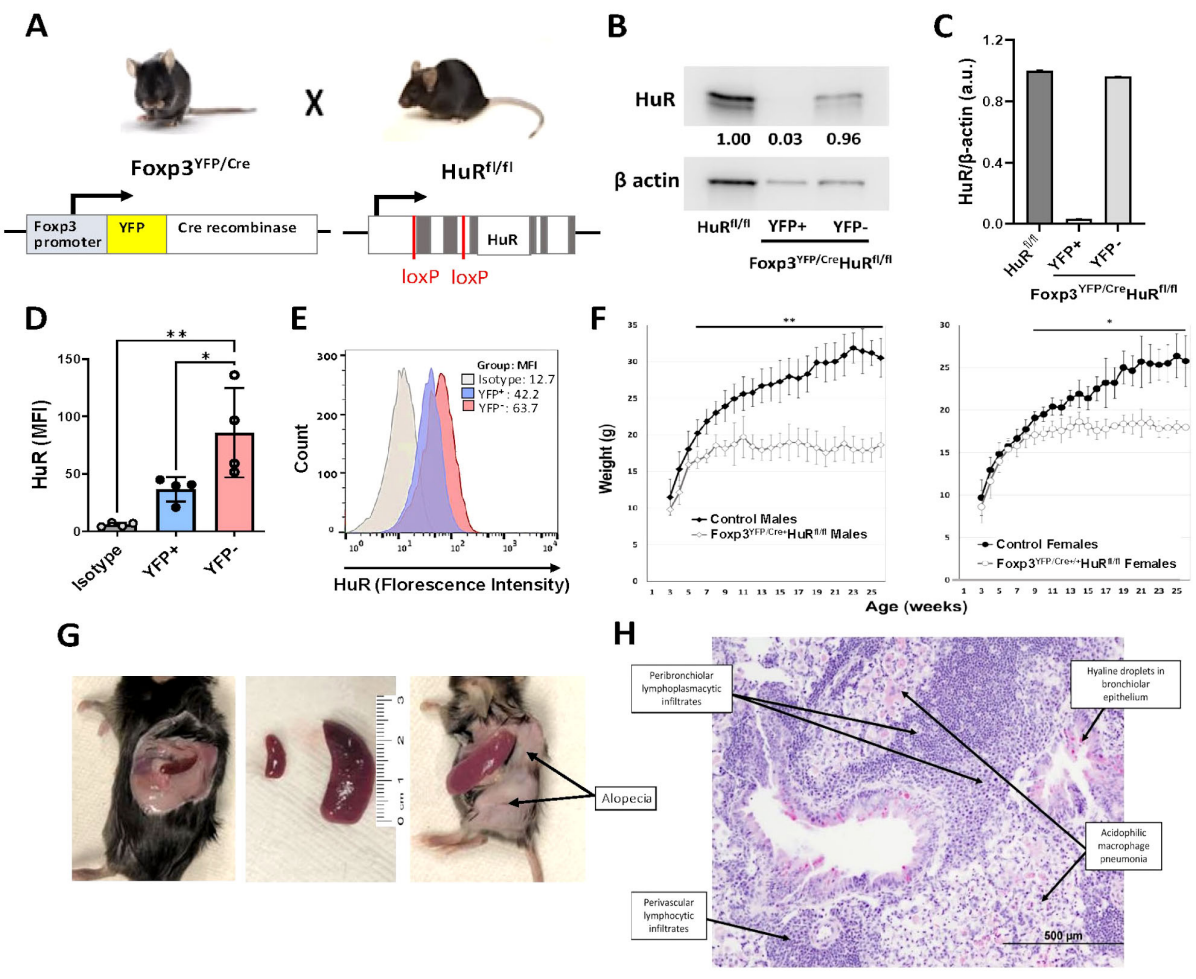
Treg-specific ablation of HuR in Foxp3*^YFP/Cre^* HuR*^fl/fl^* mice results in severe failure to thrive (FTT) and systemic spontaneous autoimmunity. **(A)** Schematic illustrating conditional deletion of HuR in Tregs in the Foxp3*^YFP/Cre^*, HuR*^fl/fl^* (HuR-KO Tregs) mouse model. **(B)** Western blot of HuR protein in sorted YFP^+^ (HuR-null) versus YFP^−^ Treg populations showing near-complete HuR deletion in YFP^+^ cells, with β-actin as loading control. **(C)** Quantification of HuR/β-actin protein levels from western blots, showing HuR reduction in YFP^+^ Tregs compared to controls. **(D)** Bar graph of HuR mean fluorescence intensity (MFI) in YFP^+^ versus YFP^−^ Tregs and isotype control, showing significant HuR reduction in YFP^+^ Tregs (mean ± SD, *p* < 0.05 by unpaired t-test, *n* = 4). **(E)** Flow cytometry histogram representative showing HuR expression profiles in YFP^+^, YFP^−^, and isotype control populations. **(F)** Body weight monitored from 3–26 weeks in male and female Foxp3*^YFP/Cre^*, HuR*^fl/fl^* (open symbols) versus controls (solid symbols). Both male and female HuR-deficient mice exhibited severe growth impairment (males plateaued at 18.5 ± 1.2 g vs. 31.5 ± 1.6 g in controls, *p* < 0.01; females plateaued at 18.3 ± 1.0 g vs. 26.5 ± 2.4 g in controls, *p* < 0.05). Two-way repeated measures ANOVA showed significant genotype effect. **(G)** Clinical phenotype in Foxp3*^YFP/Cre^*, HuR*^fl/fl^* mice including splenomegaly, alopecia, and tail stippling. **(H)** Representative H&E lung section from Foxp3*^YFP/Cre^*, HuR*^fl/fl^* mice demonstrating perivascular and peribronchiolar lymphoplasmacytic infiltrates, acidophilic macrophage pneumonia, and hyaline droplets in bronchiolar epithelium. Representative data are shown from at least 8 mice for panels F-H. The age of the mice at the time of analysis was 6–7 months. *p<0.05, **p<0.01.

The generation of the Foxp3*^YFP/Cre^* HuR*^fl/fl^*, also called HuR-KO Tregs, mice in our laboratory is described in **Figure 1A**. In brief, HuR*^fl/fl^* mice, previously generated in our lab (28), were crossed to Foxp3*^YFP/Cre^* mice (Jackson Laboratory, Bar Harbor, ME) to generate Foxp3*^YFP/Cre^* HuR*^fl/fl^* mice. HuR ablated Foxp3 cells in these mice are labeled with YFP. All mice used were on a C57BL/6 background. All animal procedures were performed under the U.S. National Institutes of Health guidelines and were approved by the University of Michigan Committee on the Use and Care of Animals. Foxp3-GFP mice (Jackson Laboratory) or HuR*^fl/fl^* mice were used as control mice where appropriate.

### 2.2 Western blot analysis of HuR expression

CD4^+^CD25^+^ cells were first enriched from the pooled spleens of at least five Foxp3*^YFP/Cre^* HuR*^fl/fl^* mice using magnetic separation with the CD4^+^CD25^+^ Regulatory T Cell Isolation Kit (Miltenyi Biotec). The enriched population was then subjected to flow cytometric sorting to separate YFP^+^ (Foxp3-expressing Tregs) and YFP^−^ cells. Sorted cells were lysed in RIPA buffer, and protein extracts were analyzed by SDS-PAGE followed by transfer to PVDF membranes. Membranes were probed with anti-HuR antibodies (Proteintech) to assess HuR expression and confirm the efficiency of HuR deletion specifically in Foxp3*^YFP/Cre^* HuR*^fl/fl^* Tregs.

### 2.3 Histological analysis

Tissue samples from various organs, including the lung, spleen, liver, and skin, among others, were collected from HuR-KO Treg mice and their wild-type (WT) controls after perfusion of the blood circulation with PBS. The tissues were fixed in 10% formalin, embedded in paraffin, sectioned, stained with hematoxylin and eosin (H&E), and evaluated for immune cell infiltration.

### 2.4 mRNA stability assay using actinomycin D

RNA stability of *Foxp3* mRNA was assessed in splenic Tregs from WT control (non-KO littermate) and Foxp3*^YFP/Cre^* HuR*^fl/fl^* (HuR-KO Tregs) mice. Tregs were isolated using the CD4^+^CD25^+^ Regulatory T Cell Isolation Kit (Miltenyi Biotec) following the manufacturer’s protocol. The isolated cells were cultured in RPMI 1640 complete medium containing 10% FBS, 2 mM L-glutamine, 1 mM sodium pyruvate (all from Gibco), 50 µg/ml gentamicin sulfate (IBI Scientific), and 0.05 mM 2-mercaptoethanol (Fisher). Cells were plated on plates pre-coated with anti-CD3 (5 µg/ml) and anti-CD28 (2 µg/ml) antibodies (Invitrogen) and activated for 4 days. On day 4, transcription was inhibited by adding actinomycin D (5 µg/ml; Sigma-Aldrich), and cells were harvested at different time points over a 4-hour period for RNA extraction. Total RNA was isolated, converted to cDNA, and analyzed by quantitative real-time PCR (qRT-PCR) to measure Foxp3 expression. The relative expression of *Foxp3* mRNA was quantified using the ΔΔCT method, normalized to *Gapdh*, with baseline (0 h) values set to 100%. In experiments involving pharmacological inhibition of HuR, WT Tregs were pretreated with either KH-3 (5 µg/ml), a small-molecule HuR inhibitor, or its inactive analog KH-3B (5 µg/ml), for 2 hours prior to activation. After 4 days of activation, cells were treated with actinomycin D as described above, and *Foxp3* mRNA stability was evaluated over 3 hours. Decay curves were plotted on a semi-log scale, and mRNA half-lives were calculated using best-fit values.

### 2.5 RNA-sequencing and pathway analysis

YFP^+^ Tregs from Foxp3*^YFP/Cre^* HuR*^fl/fl^* mice were isolated by flow cytometric sorting following magnetic enrichment for CD4^+^CD25^+^ cells from at least six individual mice. Post-sort validation by intracellular Foxp3 staining showed around 90% positivity (data not shown). RNA was isolated using standard Trizol reagent procedures and subsequently sequenced on an Illumina HiSeq platform, as previously described by our group (25). The libraries were designed to achieve a depth that allowed comprehensive coverage of all expressed genes. RNA-seq data were analyzed and validated using quality control standards (25), with a focus on capturing comprehensive gene expression profiles. Differentially expressed genes were identified based on stringent criteria: a log fold change (logFC) greater than ±1 and an adjusted p-value threshold of less than 0.05. The details of these genes, including their fold changes and statistical significance, are listed in **Supplementary Table 1**.

We performed pathway enrichment analysis using Ingenuity Pathway Analysis (IPA). To further explore gene interactions, we used the STRING database via the stringApp plugin in Cytoscape (version 3.x), incorporating both known and predicted PPIs (39, 40). This approach helped us identify key regulatory nodes and build an interaction network among significantly altered genes. Given its essential role in preserving Treg identity and function, FOXP3 was selected as a central focus in our network analysis. To ensure interaction reliability, we applied a confidence score threshold greater than 0.4.

### 2.6 Quantitative PCR assay

RNA was extracted from isolated Tregs using the RNeasy RNA Extraction Kit (Qiagen) following the manufacturer’s protocol. cDNA synthesis was carried out using SuperScript III Reverse Transcriptase (Invitrogen) according to the manufacturer’s instructions. Quantitative RT-PCR was then performed using Platinum SYBR Green Universal Master Mix (Invitrogen) on a QuantStudio 3 Real-Time PCR System (Applied Biosystems, Foster City, CA), as previously described (27, 41). Gene expression data were analyzed using the ΔΔCT method to calculate fold changes relative to the *Gapdh* as the reference gene. The primers used for amplification (Integrated DNA Technologies) are listed in **Table 1**.

**Table 1:** Sequences of mouse qPCR primers used in the study.

Gene name	Forward (5’->3’)	Reverse (5’->3’)
*Foxp3*	CTCGCATGTTCGCCTACTTC	CTCTCCACTCGCACAAAGCA
*Cd4*	CCCAGGTCTCGCTTCAGTTT	GGGAGAGGTAGGTCCCATCA
*Il2ra*	CAGACATGCAGAAGCCAACAC	AACACTCTGTCCTTCCACGA
*Rorc*	GTGGAGTTTGCCAAGCGGCTTT	CCTGCACATTCTGACTAGGACG
*Il23r*	GTCCACCAAACTTCCCAGACAG	CCTGAAGCAGGATGTCCTCTGA
*Ctla4*	GCCTTCTAGGACTTGGCCTT	CACCACTGAAGGTTGGGTCA
*Tnfrsf18*	AACGGAAGTGGCAACAACAC	CTGGTTGGCAGGGGTAGTG
*Il17a*	CAGACTACCTCAACCGTTCCAC	TCCAGCTTTCCCTCCGCATTGA
*Tgfb1*	TGATACGCCTGAGTGGCTGTCT	CACAAGAGCAGTGAGCGCTGAA
*Il10*	CGGGAAGACAATAACTGCACCC	CGGTTAGCAGTATGTTGTCCAGC
*Stat3*	AGGAGTCTAACAACGGCAGCCT	GTGGTACACCTCAGTCTCGAAG
*Gapdh*	CATCACTGCCACCCAGAAGACTG	ATGCCAGTGAGCTTCCCGTTCAG
*Actb*	TGTGATGGTGGGAATGGGTCAGAA	TGTGGTGCCAGATCTTCTCCATGT

### 2.7 RNA immunoprecipitation

RIP was performed on Tregs isolated from WT mice to investigate the interaction of HuR with its mRNA targets. The procedure followed protocols detailed in our previous publications (25, 42, 43). Tregs were isolated and pooled from at least 4 mice per experiment to ensure sufficient cell numbers. The cells were activated *in vitro* in complete RPMI for 4 days using anti-CD3 (5 µg/ml) and anti-CD28 (2 µg/ml) antibodies from eBioscience™. For the RIP assay, lysates were prepared in polysomal lysis buffer. HuR-associated RNA-protein complexes were immunoprecipitated using an anti-HuR antibody coupled to Protein A/G Sepharose beads. Normal IgG1 was used as an isotype control. RNA was extracted from the immunoprecipitates and analyzed by qRT-PCR. *β-actin*, a known HuR mRNA target, was used as a positive control, and enrichment of *Foxp3* mRNA was quantified. For each RIP experiment, pooled Tregs from four mice were treated as a single biological sample for the immunoprecipitation, and three independent pooled replicates were performed; therefore, statistical testing was not applied to individual mice.

### 2.8 Treg suppression assay

To assess suppressive function, WT or Foxp3*^YFP/Cre^* HuR*^fl/fl^* (HuR-KO Tregs) mice were either left untreated or exposed to UVB light (100 mJ/day) for five consecutive days. On Day 5, spleens were harvested, and single-cell suspensions were prepared under sterile conditions by passing the mashed tissue through 70 µm cell strainers (Falcon), followed by centrifugation and resuspension in complete HT-2 medium (DMEM supplemented with 10% FBS, 1% L-glutamine, 1% sodium pyruvate, 0.1% gentamicin, and 50 µM β-mercaptoethanol).

CD4^+^CD25^+^ regulatory T cells (Tregs) were isolated from UV-treated mice, and CD4^+^CD25^−^ effector T cells (Teffs) were isolated from non-UV-treated WT mice using magnetic separation (Miltenyi Biotec). Teffs were labeled with 5 µM Cell Proliferation Dye eFluor™ 670 (Invitrogen) by incubating for 10 minutes at 37 °C in the dark, followed by quenching with cold HT-2 medium, three washes, and resuspension at the desired concentration. In parallel, Tregs were labeled with 5 µM CellTrace™ CFSE (Invitrogen) by incubation for 20 minutes at 37 °C, then quenched with excess HT-2 medium, washed, and resuspended for co-culture.

Labeled Teffs and Tregs were co-cultured at indicated ratios (as described in the figure legend) in 96-well round-bottom plates with Dynabeads™ Mouse T-Activator CD3/CD28 (Gibco) in a final volume of 250 µL per well. Cultures were incubated for 72 hours at 37 °C in 5% CO_2_. After stimulation, cells were harvested and analyzed by flow cytometry. Teff proliferation was quantified using FlowJo software (Tree Star, Ashland, OR).

### 2.9 Statistical analyses

For statistical analysis, GraphPad Prism version 10 software (GraphPad Software, La Jolla, CA) was used. All values are expressed as means ± standard deviation. Data were analyzed by two-tailed Student *t-test*, one-way ANOVA with Tukey’s multiple comparison test, two-way repeated measures ANOVA or two-way ANOVA with Šídák’s multiple comparisons test as appropriate. Differences were considered significant when *p < 0.05*.

## 3 Results

### 3.1 Treg-specific ablation of HuR in Foxp3*^YFP/Cre^* HuR*^fl/fl^* mice results in a significant failure to thrive and spontaneous systemic autoimmunity

Our previous investigations using a T cell-specific HuR knockout (distal-Lck-Cre HuR*^fl/fl^*) revealed a significant reduction in both the frequency and expression levels of Foxp3 in splenic CD4^+^CD25^+^ T cells (data not shown). To extend these findings, we generated Foxp3*^YFP/Cre^* HuR*^fl/fl^* mice to investigate HuR’s role in maintaining Treg stability and function. These mice were produced by crossing our previously established HuR*^fl/fl^* strain (28) with the Foxp3*^YFP/Cre^* mouse (**Figure 1A**), resulting in HuR-deficient Tregs marked by YFP expression.

To further validate HuR deletion, HuR protein levels in sorted YFP^+^ and YFP^−^ Treg populations were analyzed by Western blot. As shown in **Figure 1B** and quantified in **Figure 1C**, YFP^+^ Tregs from HuR-deficient mice exhibited nearly complete loss of HuR protein, whereas YFP^−^ Tregs retained HuR levels comparable to controls. Additionally, flow cytometry studies of sorted YFP^+^ and YFP^−^ Tregs from four individual mice confirmed significant HuR reduction in the YFP^+^ population (**Figure 1D**), with representative plots shown for isotype control, WT, and KO Tregs in **Figure 1E**. These results confirm efficient and specific deletion of HuR in Tregs.

Body weight was monitored weekly from 3 to 26 weeks in male and female Foxp3*^YFP/Cre^* HuR*^fl/fl^* (KO) mice (open symbols) and littermate controls (solid symbols). Both homozygous Foxp3*^YFP/Cre^* HuR*^fl/fl^* female mice and hemizygous male mice developed failure to thrive starting around 6 weeks of age, with significantly impaired weight gain compared to controls. In males, KO mice plateaued at 18.5 ± 1.2 g by week 9, while control males reached 31.5 ± 1.6 g by week 25. In females, KO mice plateaued at 18.3 ± 1.0 g by week 14, compared to 26.5 ± 2.4 g in controls. Two-way repeated measures ANOVA revealed a significant genotype effect across the time course (males *p* < 0.01; females *p* < 0.05), with Sidak’s *post hoc* test confirming differences at multiple time points (**Figure 1F**). These results indicate that HuR deficiency in Foxp3^+^ Tregs impairs normal growth in both sexes. Foxp3*^YFP/Cre^* HuR*^fl/fl^* mice also exhibited splenomegaly, hair loss, and tail stippling (**Figure 1G**). In contrast, heterozygous Foxp3*^YFP/Cre+/-^* HuR*^fl/fl^* mice appeared phenotypically normal and were indistinguishable from WT controls (data not shown).

Additionally, Foxp3*^YFP/Cre^* HuR*^fl/fl^* mice displayed moderate to severe inflammation across multiple organs, including the kidneys, lungs, skin, stomach, liver, and spleen, as summarized in **Supplementary Table 2**, with scoring performed by a blinded board-certified veterinary pathologist. Notably, the lungs showed dense perivascular and peribronchiolar lymphoplasmacytic infiltrates, acidophilic macrophage pneumonia, and hyaline droplets in the bronchiolar epithelium (**Figure 1H**). These features are consistent with widespread immune activation and resemble the autoimmune pathology seen in scurfy mice lacking functional Foxp3. In contrast, control lung sections from healthy WT control mice showed no lesions and preserved normal histological architecture (data not shown).

### 3.2 HuR directly interacts with *Foxp3* mRNA and controls its steady-state levels and mRNA stability

**Figure 2:**
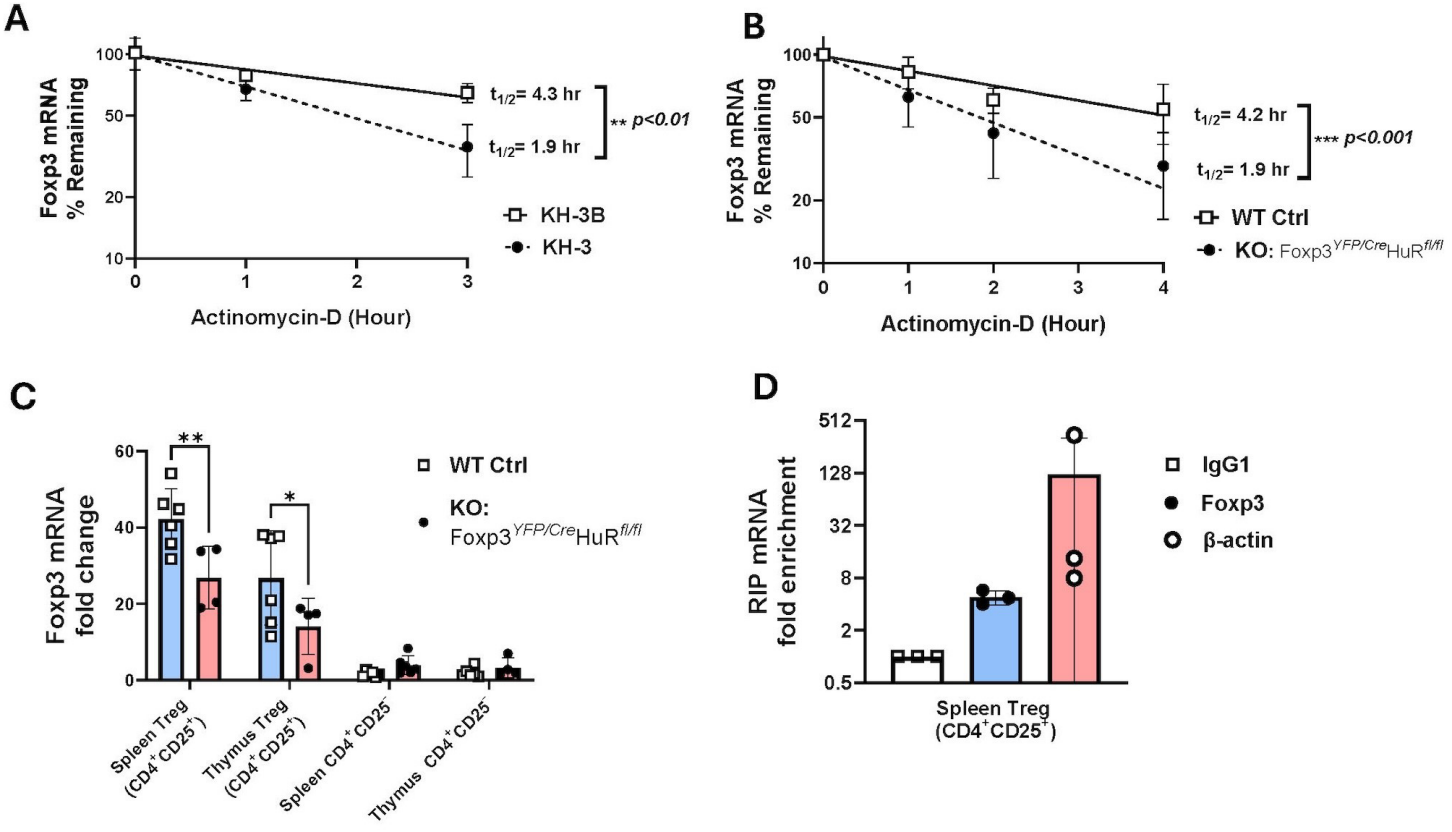
HuR directly interacts with *Foxp3* mRNA and controls its steady-state levels and mRNA stability. **(A)** *Foxp3* mRNA stability was evaluated in splenic Tregs (CD4^+^CD25^+^) isolated by magnetic column separation from WT mice treated with KH-3 or sham control (KH3-B) for 2 hours, followed by actinomycin **(D)** HuR inhibition reduced Foxp3 mRNA half-life (1.9 hours with KH-3) compared to control (4.3 hours). Data represents seven WT mice. **(B)** *Foxp3* mRNA stability was assessed in column-isolated splenic Tregs from WT and Foxp3*^YFP/Cre^* HuR*^fl/fl^* (HuR-KO Tregs) mice. HuR-KO Tregs showed a shorter Foxp3 mRNA half-life (1.9 hours) compared to WT (4.2 hours). Data represents seven WT and four HuR-KO mice. **(C)** Steady-state Foxp3 mRNA levels were measured in splenic and thymic Tregs. Foxp3 mRNA was significantly lower in HuR-KO Tregs compared to WT control, while CD4^+^CD25^−^ cells showed minimal expression with no significant differences. Data represent three HuR-KO and three WT mice. **(D)** RNA immunoprecipitation (RIP) from WT splenic Tregs showed Foxp3 mRNA enrichment in HuR pull-downs compared to IgG1 control, with β-actin as a positive control. Each point represents pooled cells from four WT mice. A total of 12 mice were used across three independent experiments. **p<0.01, ****p<0.0001.

To evaluate whether HuR regulates *Foxp3* mRNA stability, we conducted actinomycin D chase assays. WT Tregs treated with the small-molecule HuR inhibitor KH-3 exhibited a significant reduction in *Foxp3* mRNA stability, with a half-life of 1.9 hours compared to 4.3 hours in sham control-treated cells using its inactive analogue component (KH-3B, p < 0.01) (**Figure 2A**). A similar analysis in HuR-KO Tregs (*Foxp3^YFP/Cre^ HuR^fl/fl^*) revealed a substantial reduction in *Foxp3* mRNA stability compared to WT control Tregs, with half-lives of 1.9 and 4.2 hours, respectively (p < 0.001) (**Figure 2B**). These results indicate that HuR stabilizes *Foxp3* mRNA and contributes to maintaining its steady-state levels. Quantification of *Foxp3* steady-state mRNA levels in splenic and thymic Tregs further underscores HuR’s essential role in maintaining *Foxp3* expression, as *Foxp3* mRNA was markedly reduced in KO compared to WT (p < 0.01 in spleen and p < 0.05 in thymus) (**Figure 2C**). This reduction was specific to the CD4^+^CD25^+^ Treg subset, as CD4^+^CD25^−^ cells exhibited negligible *Foxp3* mRNA levels, irrespective of HuR expression. Together, these findings identify HuR as a key post-transcriptional regulator of *Foxp3* mRNA in Tregs. RIP studies revealed enrichment of *Foxp3* mRNA in the HuR pull-down fraction, showing around 8-fold increase compared to IgG1 controls. As expected, the positive control *β-actin* mRNA, due to relative abundance of transcript, exhibited high enrichment, validating the RIP assay (**Figure 2D**). As described in the Methods, RIP experiments were performed on pooled Treg samples, which were treated as single measurements; therefore, formal statistical comparisons were not feasible. These findings demonstrate that HuR directly interacts with *Foxp3* mRNA, providing a mechanistic basis for its role in regulating mRNA stability and steady-state levels.

### 3.3 Gene profiling of RNA-seq and PPI analysis reveals direct interaction between Rorc and Foxp3 in HuR-KO Tregs

To explore the downstream consequences of HuR ablation in Tregs, we performed RNA sequencing (RNA-Seq) on YFP^+^ Tregs isolated from *Foxp3^YFP/Cre^ HuR^fl/fl^* (HuR KO) mice and WT controls. This analysis identified significant transcriptional dysregulation in the HuR-KO Treg population, including key molecules involved in T-helper cell differentiation (**Supplementary Table 3**).

**Figure 3:**
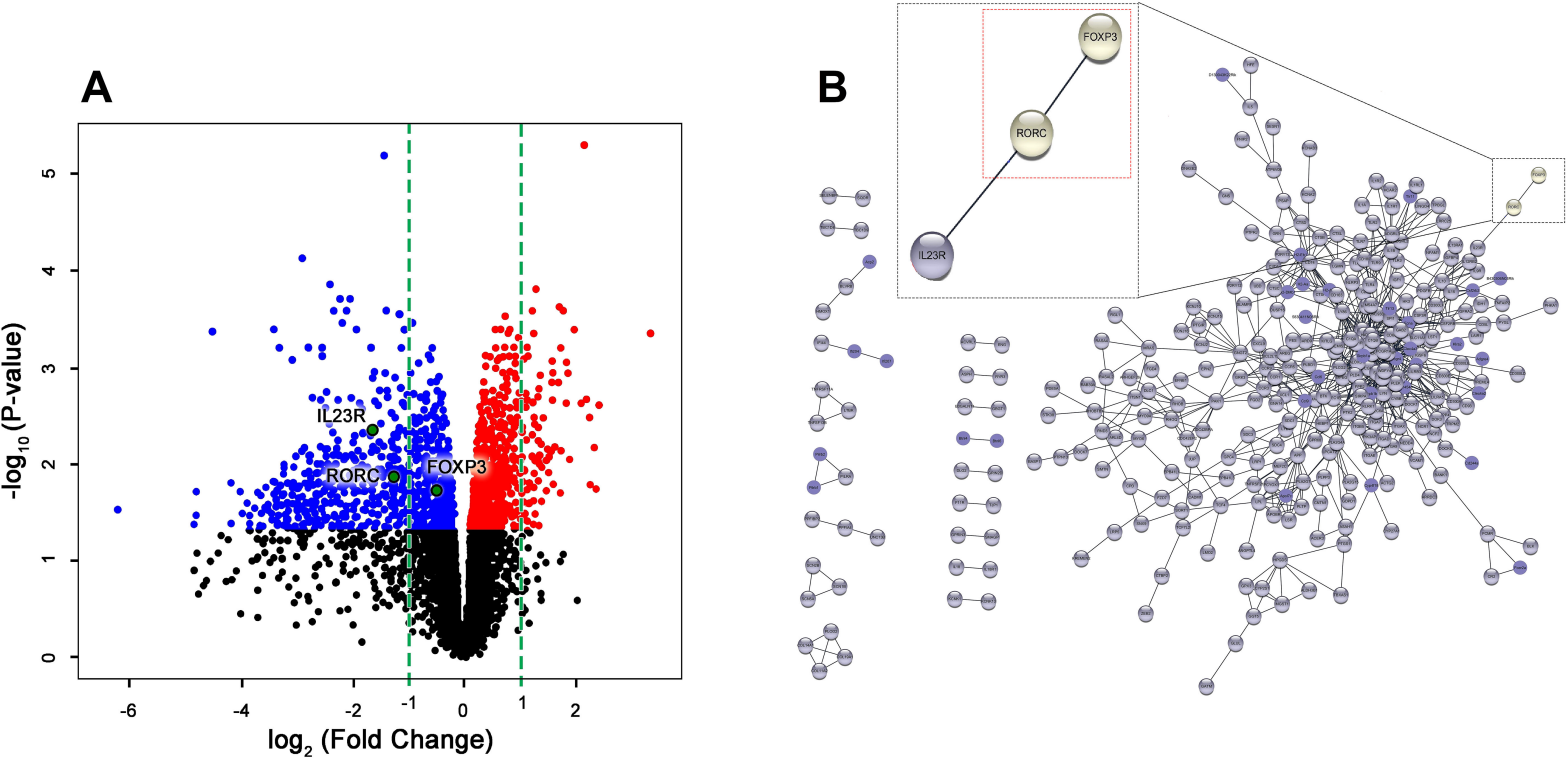
Gene profiling of RNA-seq and PPI analysis reveals direct interaction between *Rorc* and *Foxp3* in HuR-KO Tregs in Foxp3*^YFP/Cre^* HuR*^fl/fl^* Tregs. **(A)** Volcano plot visualization of RNA-Seq results from sorted YFP^+^ (HuR-KO Tregs) isolated from the spleen of Foxp3*^YFP/Cre^* HuR*^fl/fl^* mice. The plot shows the expression levels of 2220 statistically significant genes in YFP^+^ (HuR-KO Tregs), with differentially expressed genes highlighted (upregulated in red, downregulated in blue). *FOXP3*, Rorc, and *IL-23R*, were identified and labeled. **(B)** Protein-protein interaction (PPI) network analysis using Cytoscape identified significant gene interactions. Among the differentially expressed genes in YFP^+^ (HuR-KO Tregs), Rorc (RORγt) was found to directly interact with FOXP3, as shown in the enlarged region (red dashed box). n = 6 mice.

RNA-Seq analysis identified 2,220 significantly dysregulated genes in YFP^+^ Tregs from HuR-KO Tregs mice compared to WT controls (**Supplementary Table 1**). The volcano plot highlights differentially expressed genes, with the top genes visualized (**Figure 3A**). Among these, *Foxp3* itself was significantly downregulated, suggesting the direct regulatory role of HuR in stabilizing *Foxp3* mRNA. Similarly, *Rorc* (encoding *RORγt*) and *Il23R* were also significantly downregulated, suggesting a perturbation in the Treg-Th17 axis. This axis is critical for maintaining the balance between regulatory and effector T cell functions, and its dysregulation could contribute to the autoimmune phenotype observed in HuR-KO Tregs mice.

To further explore the molecular pathways disrupted in HuR-deficient Tregs, Ingenuity Pathway Analysis (IPA) was performed on the RNA-Seq data. This analysis identified significant enrichment of genes involved in T helper cell differentiation, with a specific focus on the interplay between *Foxp3* and *Rorc*. **Supplementary Table 3** illustrates the involvement of *Rorc* in the differentiation of Th17 cells, a key effector T cell subset that maintains immune balance alongside Tregs. The role of *Foxp3* and *Rorc* in opposing cellular fates is further emphasized in the pathway. As shown in the enlarged region of **Supplementary Table 3**, *Foxp3* suppresses *Rorc* to promote Treg identity, while *Rorc* promotes Th17 differentiation. The simultaneous downregulation of both *Foxp3* and Rorc in HuR-deficient Tregs suggests a breakdown in this tightly regulated axis.

PPI analysis further identified a direct interaction between Foxp3 and Rorc (**Figure 3B**), supporting the hypothesis that HuR plays a critical role in maintaining Treg function by regulating the Treg-Th17 axis. The downregulation of *Rorc* and *Il23r* in HuR-KO Tregs suggests a potential mechanism through which HuR regulates immune tolerance and contributes to the autoimmune phenotype observed in the model.

### 3.4 Altered mRNA expression of selected genes involved in T helper differentiation and Treg function in Foxp3*^YFP/Cre^* HuR*^fl/fl^* Tregs compared to WT control Tregs

**Figure 4:**
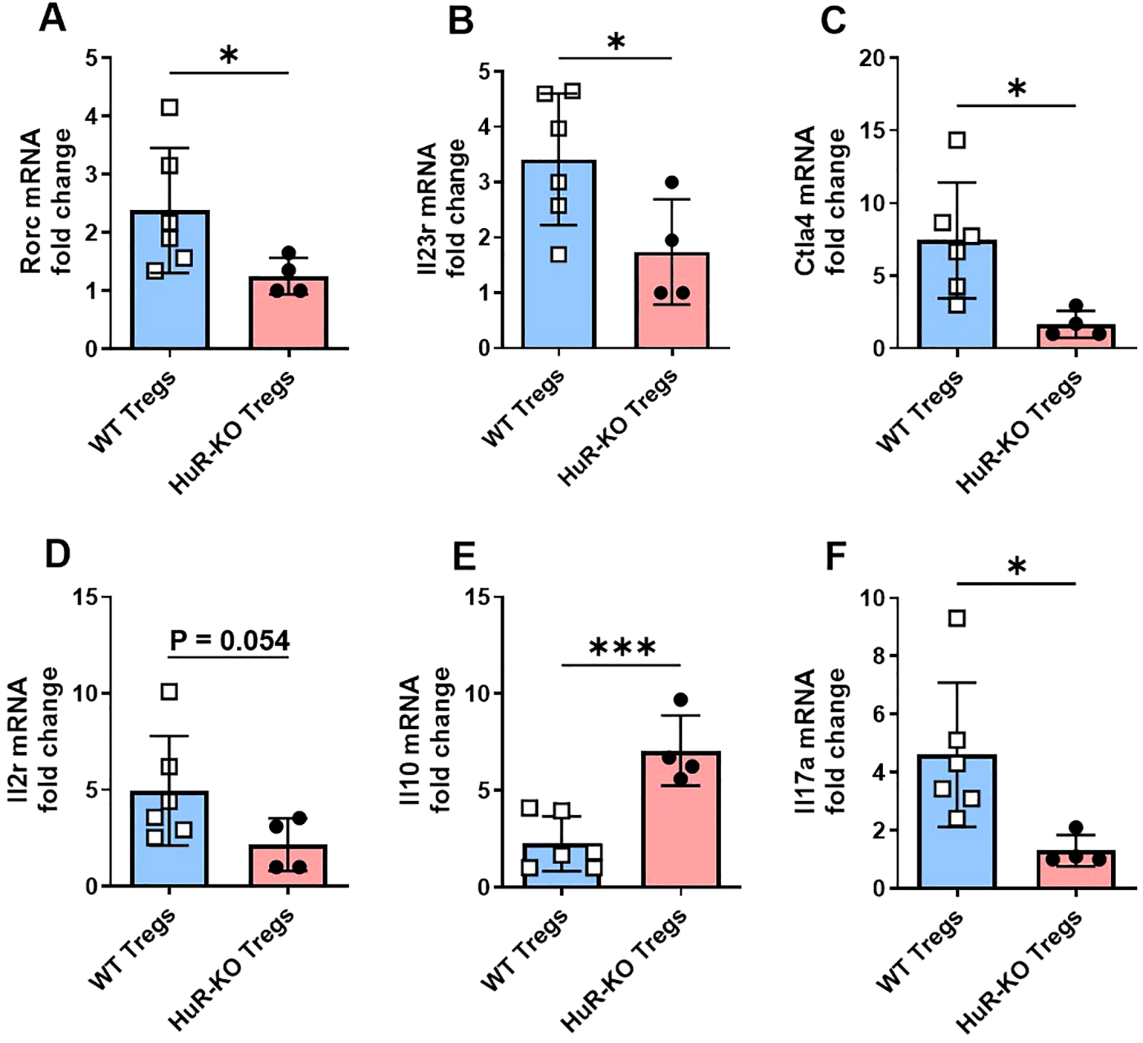
Altered mRNA expression of selected genes involved in T helper differentiation and Treg function in Foxp3*^YFP/Cre^* HuR*^fl/fl^* Tregs compared to WT control Tregs. **(A-F)** mRNA expression of key genes involved in T helper differentiation (based on RNA-seq IPA analysis) and Treg function including Rorc, Il23r, Ctla4, Il2r, Il10 and Il17a was measured by qPCR in CD4^+^CD25^+^ Tregs isolated from the spleens of HuR-KO and WT control mice, following 4 days of activation with anti-CD3 and anti-CD28. Data were analyzed using unpaired Student’s *t*-test. Results are representative of four independent HuR-KO mice and a total of six corresponding WT controls, from cohorts distinct from those used in RNA-seq analysis.

The mRNA quantification of selected genes involved in Treg function and T helper differentiation pathways in *Foxp3^YFP/Cre^ HuR^fl/fl^* (HuR-KO Tregs) compared to WT control Tregs is shown in **Figure 4**. The mRNA levels of *Rorc* (**Figure 4A**), which encodes *RORγt* and is critical for Th17 differentiation, were significantly reduced in HuR-KO Tregs compared to WT Tregs (*p < 0.05*). Similarly, *Il23r* (**Figure 4B**), a receptor involved in Th17 differentiation, also showed a significant decrease in HuR-KO Tregs (*p < 0.05*), further highlighting the dysregulation of the Treg-Th17 balance. The mRNA expression of *Ctla4* (**Figure 4C**), an important molecule for Treg-mediated immune suppression, was also significantly reduced in HuR-KO Tregs. *Il2r* (encoding CD25) (**Figure 4D**), a key receptor for IL-2 signaling in Tregs, showed downregulated levels, though the result approached significance in HuR-KO Tregs (*p = 0.054*). The mRNA levels of *Il10* (**Figure 4E**), an anti-inflammatory cytokine, were significantly increased in HuR-KO Tregs (*p < 0.001*). Additionally, *Il17a* (**Figure 4F**), a pro-inflammatory cytokine associated with Th17 cells, was significantly downregulated in HuR-KO Tregs (*p < 0.05*), further supporting the disruption of the Treg-Th17 axis. Genes such as *Tnfrsf18* (encoding GITR), *Stat3*, and *Tgfb1*, were also assessed but did not show significant differences between the two groups (data not shown). These results suggest that the disruption of HuR leads to impaired genes of Treg function and those involved in the T helper differentiation pathway, particularly affecting the balance between Treg and Th17 cell function.

### 3.5 Impaired suppressive function of HuR KO-Tregs (Foxp3*^YFP/Cre^* HuR*^fl/fl^*) on T effector cell proliferation

We performed an immunosuppression assay to evaluate the suppressive capacity of Tregs isolated from the spleens of Foxp3*^YFP/Cre^* HuR*^fl/fl^* (KO) and HuR*^fl/fl^* (WT) mice. Tregs (CD4^+^CD25^+^) were co-cultured with labeled effector T cells (Teff, CD4^+^CD25^−^) at varying Treg: Teff ratios (1:2 to 1:512) and activated with anti-CD3/CD28 Dynabeads. The proliferation of Teff cells was measured and expressed as a percentage of proliferating cells.

**Figure 5:**
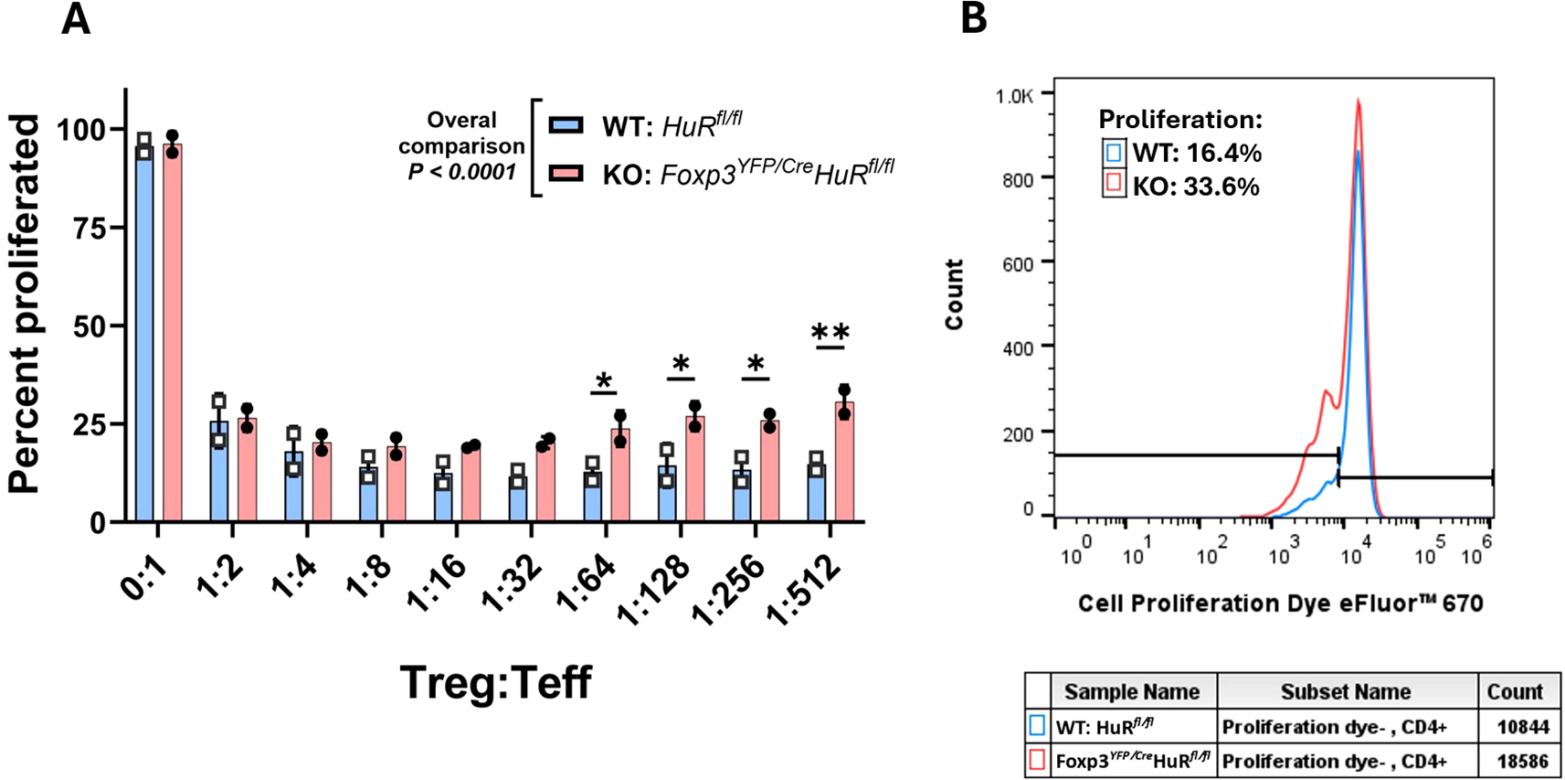
Impaired suppressive function of HuR-KO Tregs (Foxp3*^YFP/Cre^* HuR*^fl/fl^*) on Teffector cell proliferation. The *in vitro* suppressive ability of Tregs in regulating Teffector cell (Teff) proliferation was evaluated by flow cytometry. Teff cells were labeled with a proliferation dye prior to co-culture with isolated Tregs (CD4^+^CD25^+^) from control HuR*^fl/fl^* WT (blue bars) and Foxp3*^YFP/Cre^* HuR*^fl/fl^* (red bars) mice at varying Treg: Teff ratios, as indicated on the x-axis. **(A)** Percentage suppression of Teff cell proliferation by CD4^+^CD25^+^ Tregs across different Treg: Teff ratios. HuR-KO Tregs showed a significant, progressive decline in suppressive capacity beginning at the 1:64 ratio, whereas WT Tregs maintained stable suppression even at lower Treg frequencies. **(B)** Representative flow cytometry histograms of Teff proliferation patterns (gated on live cells), demonstrating reduced suppression by HuR-KO Tregs compared to WT Tregs. Overall, HuR-KO Tregs exhibited significantly reduced suppression of Teff cell proliferation (*p* < 0.0001). Data are from five mice per group, each run-in duplicate. Data were analyzed using two-way ANOVA followed by Šídák’s multiple comparisons test. *p<0.05; **p<0.01.

In **Figure 5**, panel A summarizes the Teff proliferation percentages across the different Treg: Teff ratios achieved by titrating Treg cell numbers, while panel B shows a representative flow cytometry histogram demonstrating higher Teff proliferation when co-cultured with HuR-KO Tregs compared to WT Tregs, suggesting impaired suppressive function of the HuR-KO Tregs.

Overall, HuR-deficient (KO) Tregs exhibited significantly reduced suppressive capacity compared to WT Tregs (p < 0.0001). In WT Tregs, suppression of Teff proliferation reached a plateau from 1:8 to 1:512 Treg: Teff ratios, indicating stable suppressive potential even at lower Treg frequencies. In contrast, KO Tregs showed a progressive decline in suppressive capacity starting at the 1:64 ratio, failing to achieve a comparable plateau. This pattern suggests that HuR may be important for sustaining Treg-mediated suppression under conditions of high Teff challenge and low Treg frequency, where intrinsic regulatory mechanisms, such as post-transcriptional control via HuR, may become limiting. This is conceptually consistent with previous reports highlighting Treg functional thresholds under Teff-dominant conditions (44–46).

## 4 Discussion

Our data identify a new role for HuR in stabilizing *Foxp3* mRNA, which is essential for sustaining Treg function. Using RIP and actinomycin D assays, we showed that HuR binds to *Foxp3* mRNA and stabilizes it, preventing degradation. This is in line with earlier studies on other cell types showing HuR promotes mRNA stability by binding to AREs in the 3′ UTRs of target mRNAs expanding our previous work on HuR’s post-transcriptional regulation during T cell activation (22, 25–27). Here, we specifically show that this mechanism is relevant in Tregs. By generating a novel mouse model of Foxp3*^YFP/Cre^* HuR*^fl/fl^* through conditional ablation of HuR in Tregs, we identified extensive phenotypic and molecular disruptions in Tregs, resulting in an autoimmune phenotype. One of the interesting findings was that Foxp3 expression was reduced in the HuR-KO Tregs, which occurred despite a compensatory increase in Treg populations numbers, as revealed in flow cytometry: ~15% YFP^+^ CD4^+^ cells in the spleen, compared to ~10% GFP^+^ cells in Foxp3-GFP controls (**Supplementary Figure 2A**).

As stated above, dysregulation of Foxp3 contributes to various autoimmune diseases due to uncontrolled inflammation (2–5). Given that *FOXP3* mutations cause IPEX in humans and the scurfy phenotype in mice (16, 17), it is notable that Treg-specific HuR deletion produced a similarly severe inflammatory phenotype, albeit with extended survival. However, unlike scurfy mice, they survived longer, which may make them useful for studying chronic Treg dysfunction and testing therapeutic strategies, even though they remained clinically ill throughout their life. A recent study by Rudensky’s group showed that *Foxp3* restoration in Treg-deficient mice reverse systemic autoimmunity even in established inflammation (47). Their data underscores the essential role of Foxp3 in maintaining immune tolerance and preventing autoimmune pathology. While *Foxp3* reconstitution can rescue autoimmunity (47), our findings highlight that proper regulation of *Foxp3* expression, mediated by HuR, is critical to prevent the onset of autoimmunity (48). Supporting this, deletion of miR-15/16 in Tregs similarly disrupts *Foxp3* expression and immune tolerance, underscoring the broader importance of RNA-based post-transcriptional regulation in Treg stability.

Our het females who did not show abnormal phenotype showed down regulation of *Foxp3* in their YFP^+^ population. This is similar to the female carrier for the IPEX syndrome who only express mild form of the disease.

Our RNA-seq analysis revealed significant disruption of immune regulatory pathways, particularly T helper cell differentiation, with *Foxp3* emerging as a central regulator of Treg function. HuR-deficient Tregs exhibited downregulation of both *Foxp3* and Rorc (encoding RORγt), suggesting a disturbed Treg-Th17 axis, as confirmed by RNA-seq and qPCR. We identified Rorc as a key node connecting Foxp3 to the Th17 pathway via IL-23R in our PPI network analysis, implicating these genes in the maintenance of Treg-Th17 balance. Consistent with this, IL-23R was also significantly downregulated in HuR-KO Tregs, showing a failure in this regulatory axis. An earlier study showed a similar defect in Th17 differentiation in HuR-KO CD4^+^ T cells in the spleen during experimental autoimmune encephalomyelitis (EAE), where loss of HuR in OX40-Cre HuR*^fl/fl^* mice reduced RORγt, IRF4, and RUNX1 expression, key transcription factors essential for Th17 lineage commitment, ultimately impairing Th17 responses and autoimmune neuroinflammation (30). Rorc and *Foxp3* are known to antagonize each other to dictate the balance between Tregs and Th17 cells. The simultaneous reduction of *Foxp3* and Rorc in HuR-KO Tregs suggests a disrupted regulatory interplay, which likely contributes to increased inflammatory responses. This dysregulation has been implicated in various autoimmune diseases (49–54). Our findings position HuR as an upstream regulator that maintains this axis through post-transcriptional stabilization of *Foxp3* and its interacting partners (2–5). By destabilizing *Foxp3* and its linked components, such as Rorc and *IL-23R*, HuR ablation may influence signaling pathways required for Treg differentiation and Th17 regulation. This extends our understanding of the intricate balance between Tregs and Th17 cells and highlight HuR’s role in maintaining immune balance. Although we did not investigate direct evidence of HuR binding to *Rorc* mRNA in Tregs, and to our knowledge no prior study has confirmed direct HuR-Rorc RIP in T cells, its downregulation may plausibly reflect direct HuR targeting as suggested by our previous work in other Th17-associated pathways (29–31), or indirect regulation through Foxp3-dependent transcriptional networks. Future RIP-seq or CLIP analyses using larger pooled Treg samples could help clarify whether *Rorc* is a direct HuR target within the Treg compartment.

Interestingly a recent study using bioinformatics analysis of human Crohn’s disease identified *ELAVL1* as a top upregulated hub gene in peripheral blood, with enrichment in T cell activation and NOD-like receptor signaling pathways (55), highlighting the importance role of HuR in immune dysregulation and its potential contribution to autoimmune and inflammatory disease contexts.

We found an upregulation of IL-10, a key anti-inflammatory cytokine, which may reflect a compensatory response to the heightened inflammation in HuR-KO Tregs. This is consistent with earlier studies which have shown elevated levels of IL-10 in autoimmune diseases such as SLE, RA, and MS (56), where IL-10 often acts as an ‘alarm cytokine’ connected to the disease severity and progression (57, 58). These findings show the paradoxical role of IL-10 in immune regulation which may contribute to the inflammatory responses observed in HuR-KO Tregs mice.

We found downregulation of *Il2ra* (encoding CD25) and *Ctla4* in HuR-KO Tregs. Since Treg-mediated immunosuppression is critical for controlling effector T cell proliferation and function (59–61), reduced expression of these molecules may contribute to the impaired suppressive capacity of HuR-KO Tregs. CD25, the alpha chain of the IL-2 receptor, is essential for Treg survival and function by allowing efficient uptake of environmental IL-2 during activation (62–65). In typical Tregs, CD25 is abundantly expressed and plays a key role in limiting Teff growth by depleting IL-2 from their surroundings (63). Our findings complement our previously published work which demonstrates that HuR posttranscriptionally regulates IL-2 homeostasis and CD4^+^ Th2 differentiation (26). This highlights the importance of HuR in maintaining the functional integrity of Tregs.

Lastly, based on immunophenotyping of a limited number of HuR-KO Treg mice (n = 3) (**Supplementary Figure 2**), we observed dysregulation across various immune cell populations, not restricted to Tregs. These immune changes resemble those observed in models of SLE and RA, where imbalanced inflammation and loss of self-tolerance are well-documented features (66–71). The immune phenotyping data on HuR-KO Tregs mice, although with limited mice, exhibits increase in plasmacytoid dendritic cells (pDCs) but decreased myeloid DCs. This pattern mirrors findings in human SLE, where elevated pDC levels and reduced mDC numbers are reported (72). These cells are implicated in the progression of autoimmune diseases like SLE and psoriasis (73). The altered dendritic cell profile in our model suggests that HuR may play a critical regulatory role in dendritic cell homeostasis and function, potentially offering novel insights into the molecular mechanisms driving autoimmune responses.

We acknowledge several limitations in our study to consider. 1) The number of HuR-KO mice available for experiments was limited due to poor breeding efficiency from using sick male knockouts and carrier female mice, despite optimization efforts such as modified chow and supportive care. As a result, tissue collection was often based on availability rather than tightly age-matched time points, introducing unavoidable variability that may have influenced the immune responses. 2) While we focused on the Treg-Th17 axis, Th1 responses and potential Treg plasticity toward Th1-like phenotypes may also contribute to the observed inflammation and warrant further investigation. 3) *In vivo* suppression assays, such as adoptive transfer of HuR-deficient Tregs into lymphopenic hosts, would have provided more direct evidence for their functional competence, but these experiments were not feasible in the current study due to limited Treg numbers and poor colony health in the HuR-KO mouse colony. 4) For most of our experimental studies, Tregs were isolated as CD4^+^CD25^+^ cells, which may include a small number of activated non-Tregs. 5) Finally, we did not evaluate cytokine production by Teff cells in the co-culture with Tregs due to limited cell numbers; future studies can investigate this to further define the functional consequences of HuR-KO Tregs on Teff responses.

Despite these constraints, our study offers new insights into HuR’s role in immune regulation. Loss of HuR in Tregs led to widespread immune dysregulation, implicating HuR in both Treg function and broader immune homeostasis. Using a novel Treg-specific HuR-deficient mouse model, we demonstrated that HuR binds and stabilizes *Foxp3* mRNA in Tregs *ex vivo*, providing evidence of its regulatory role in maintaining Treg function *in vivo*. Future studies involving 3′UTR luciferase reporter assays will be important to precisely define the HuR-binding regions within the Foxp3 transcript. Additionally, adoptive transfer experiments using HuR-deficient Tregs in lymphopenic hosts could directly test their *in vivo* suppressive function and further validate the physiological relevance of our findings. Since HuR (*ELAVL1*) is also among the top upregulated hub genes in blood samples from Crohn’s disease patients, underscoring its translational relevance (55). With future support, we aim to expand our colony and further investigate HuR’s role across immune compartments and autoimmune disease contexts.

## Data Availability

The datasets presented in this study can be found in online repositories. The names of the repository/repositories and accession number(s) can be found in the article/**Supplementary Material**.
