# Supplementary material for: HuR ablation destabilizes Foxp3 mRNA and impairs regulatory T cell function, contributing to an autoimmune phenotype
Source: Front Immunol. 2025 Sep 26;16:1618677. doi: 10.3389/fimmu.2025.1618677 (PMC12511036; doi:10.3389/fimmu.2025.1618677)

**Supplementary Fig. 1. RORγt (encoded by *RORC*) involves in T Helper Cell Differentiation pathway in YFP$^{+}$ HuR-KO Tregs (Foxp3$^{YFP/Cre}$ HuR$^{fl/fl}$) based on RNA-Seq analysis.**

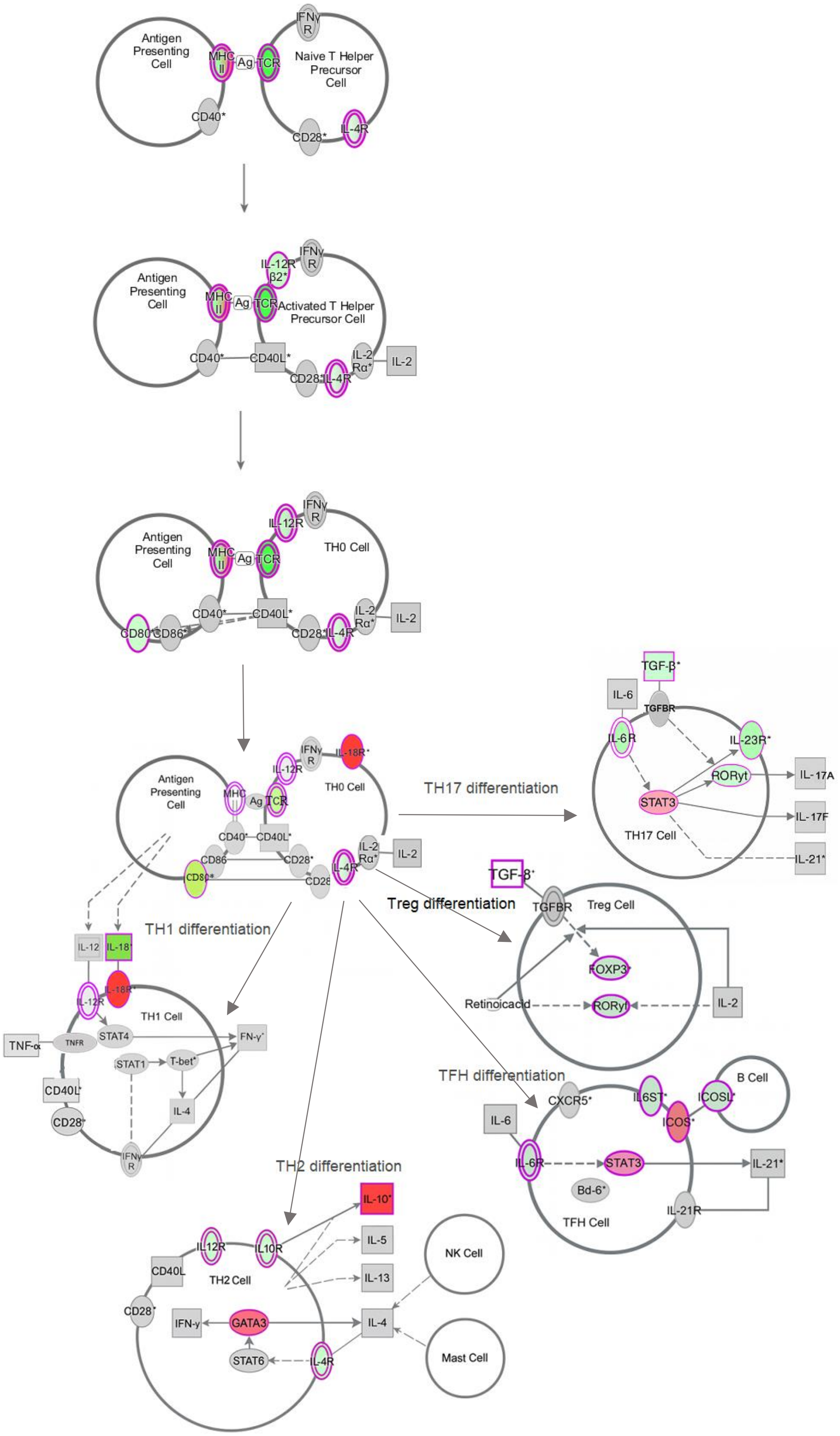

Supplement: Supplementary file 1 [file DataSheet1.pdf]
