# Supplementary material for: HuR ablation destabilizes Foxp3 mRNA and impairs regulatory T cell function, contributing to an autoimmune phenotype
Source: Front Immunol. 2025 Sep 26;16:1618677. doi: 10.3389/fimmu.2025.1618677 (PMC12511036; doi:10.3389/fimmu.2025.1618677)

Supplementary Fig. 2: Disrupted immune cell phenotype in Foxp3$^{YFP/Cre}$ HuR$^{fl/fl}$ spleen compared to wild-type control. Data representative of three mice.

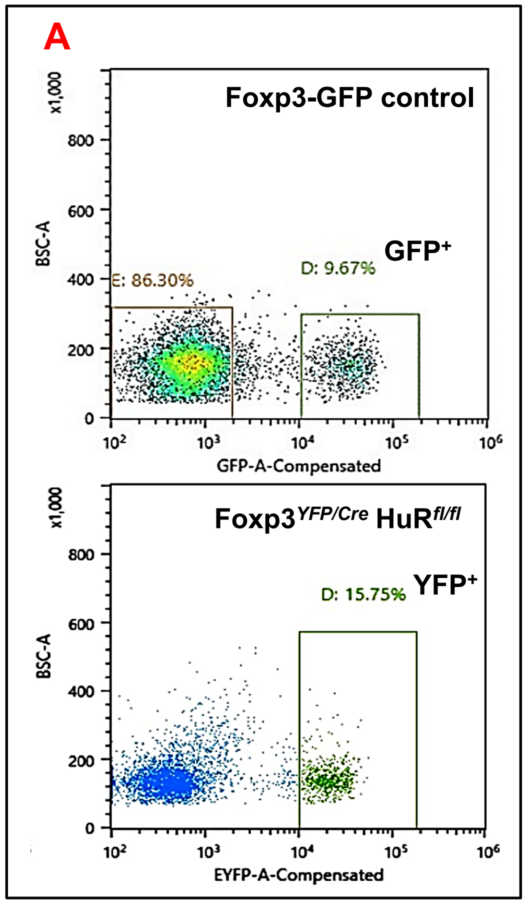

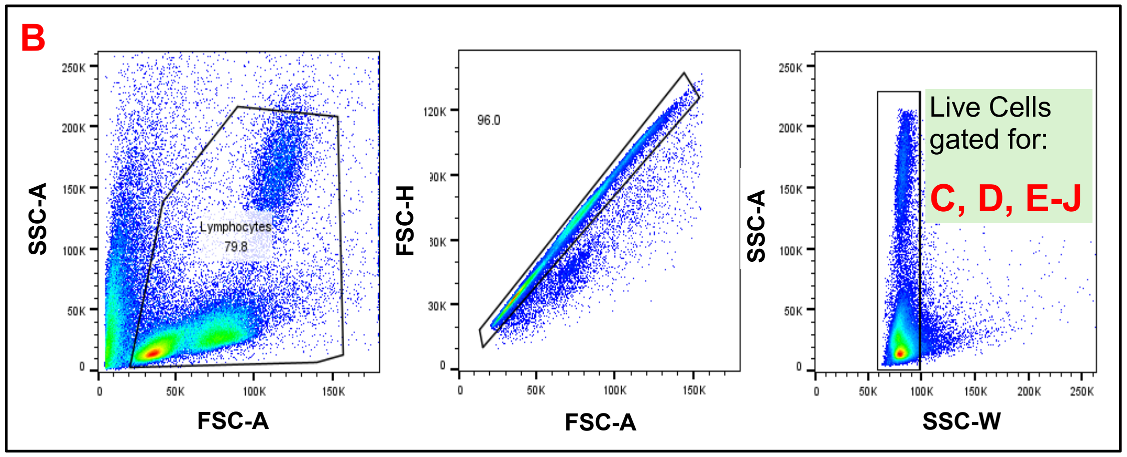

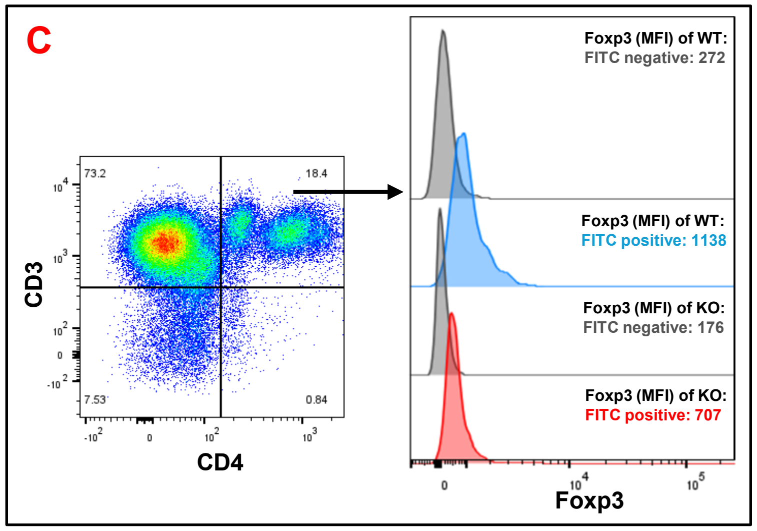

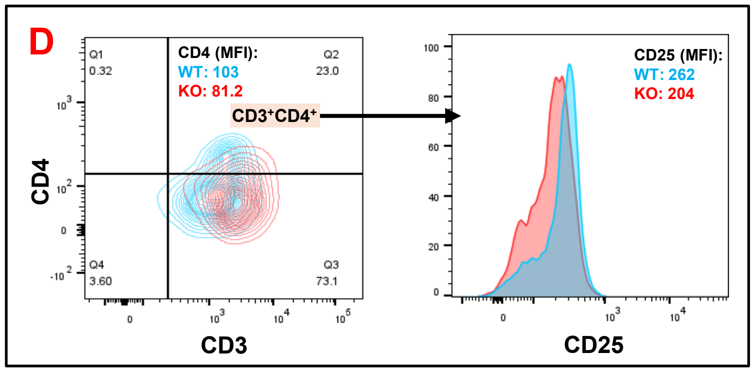

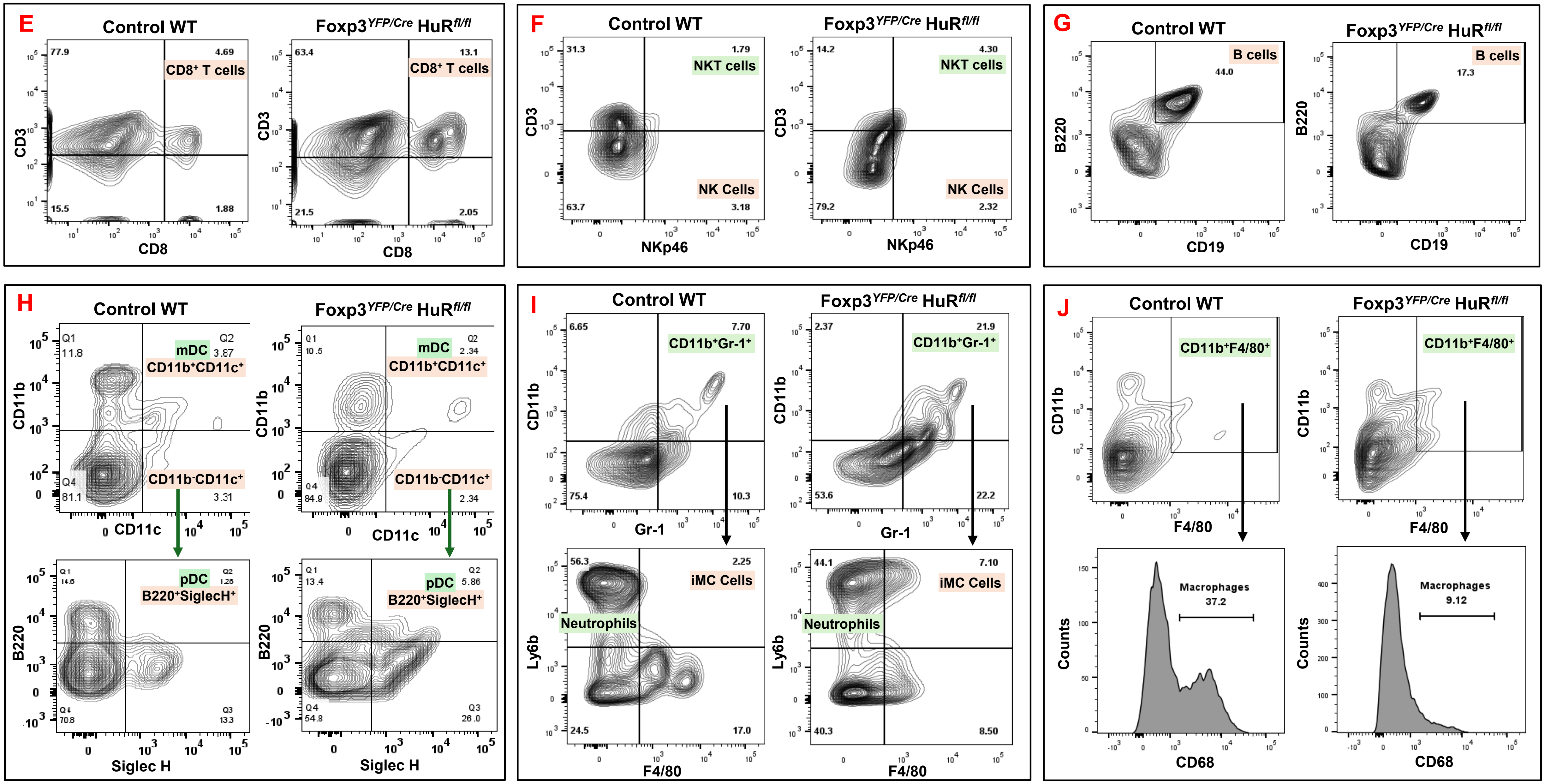

Supplement: Supplementary file 2 [file DataSheet2.pdf]
