# Supplementary material for: HuR ablation destabilizes Foxp3 mRNA and impairs regulatory T cell function, contributing to an autoimmune phenotype
Source: Front Immunol. 2025 Sep 26;16:1618677. doi: 10.3389/fimmu.2025.1618677 (PMC12511036; doi:10.3389/fimmu.2025.1618677)
Supplement: Supplementary file 3 [file DataSheet3.docx]

Supplementary Material

# Supplementary Materials and Methods

**Surface and intracellular staining for flow cytometric analysis**

For flow cytometry analysis, single-cell suspensions were prepared from lymphoid organs, including the spleen, thymus, lymph nodes, and bone marrow for immunophenotyping of HuR-KO Treg and WT mice using immune cell panels adapted from Hensel *et al* (1). Cells were harvested and processed for flow cytometry after red blood cell lysis, according to previously established protocols (2, 3). After assessing cell viability using the LIVE/DEAD™ Fixable Dead Cell Stain Sampler Kit (Invitrogen, Catalog number L34960) with the appropriate dyes that best fit the spectral profiles of the multicolor antibody panels, the live cells were then used for subsequent gating in the stained cells of each panel. The list of antibodies for each cell type characterization panel, together with the selected viability dyes, is provided in Supplementary Table 4.

Prior to staining, cells were blocked with mouse TruStain FcX™ Fc Receptor Blocking Solution (BioLegend) to minimize non-specific binding. Cells were then processed for surface staining using fluorochrome-conjugated antibodies. Surface staining was performed at 4 °C for 30 minutes in the dark. For intracellular staining of Foxp3, cells were fixed with 2% paraformaldehyde for 15 minutes at room temperature in the dark to preserve the fluorescence of YFP/GFP. After fixation, cells were permeabilized using the Foxp3/Transcription Factor Staining Buffer Set (eBioscience) following the manufacturer's instructions. Intracellular staining for Foxp3 (eFluor660, eBioscience) was performed in permeabilization buffer for 30 minutes at 4°C. Stained cells were acquired on a BD FACSCanto™ flow cytometer. Fluorescence compensation was done using UltraComp eBeads™ Plus Compensation Beads, ArC™ Amine Reactive Compensation Bead Kit, and GFP BrightComp Beads (all from Invitrogen), where appropriate, to adjust for spectral overlap among the different fluorochromes. The viability dye selection was optimized for each panel to minimize spectral spillover, and appropriate compensation controls were included to correct for any potential overlap between the viability dyes, antibody fluorochromes, and endogenous YFP fluorescence. Data were analyzed using FlowJo software (Tree Star, Ashland, OR).

**2 Supplementary figure captions**

**Supplementary Figure 1. RORC involves in T Helper Cell Differentiation pathway in YFP^+^ HuR-KO Tregs (Foxp3*^YFP/Cre^* HuR*^fl/fl^*) based on RNA-Seq analysis.** Ingenuity Pathway Analysis (IPA) of RNA-seq data from YFP^+^ HuR-KO Tregs (Foxp3*^YFP/Cre^* HuR*^fl/fl^*) illustrating the involvement of RORC in the T helper cell differentiation pathway. The pathway highlights key molecules, including Foxp3, RORC, and IL-23R, and other key molecules. Genes detected in the RNA-seq dataset are indicated within the pathway diagram.

**Supplementary Figure 2: Disrupted immune cell phenotype in Foxp3*^YFP/Cre^* HuR*^fl/fl^* spleen compared to wild-type control. A.** Sorting of isolated CD4^+^ cells from control Foxp3-GFP and Foxp3*^YFP/Cre^* HuR*^fl/fl^* mice for percentages of GFP^+^ or YFP^+^ cells, **B.** Gating strategy for splenocytes from control WT and Foxp3*^YFP/Cre^* HuR*^fl/fl^* mice to assess: **C.** CD3^+^CD4^+^ cells and subsequent Foxp3 expression (MFI) in separate FITC^+^ and FITC^-^ cells, **D.** CD4^+^CD3^+^ cells and subsequent CD25 expression (MFI), **E.** CD3^+^CD8^+^ (CD8^+^ T cells), **F.** CD3^-^NKp46^+^ (NK cells), **G.** CD19^+^B220^+^ (B cells), **H.** CD11b^+^CD11c^+^ (myeloid dendritic cells, mDCs) and B220^+^SiglecH^+^ (plasmacytoid dendritic cells, pDCs), **I.** CD11b^+^GR-1^+^ gated for Ly6b^+^F4/80^-^ (neutrophils) and Ly6b^+^F4/80^+^ (immature myeloid cells, iMCs) **J.** CD11b^+^F4/80^+^CD68^+^ (macrophages). Data are representative of three mice per group. MFI: Mean Fluorescence Intensity.

**3 References:**

1. Hensel JA, Khattar V, Ashton R, Ponnazhagan S. Characterization of immune cell subtypes in three commonly used mouse strains reveals gender and strain-specific variations. Lab Invest. 2019;99(1):93-106.

2. Techasintana P, Ellis JS, Glascock J, Gubin MM, Ridenhour SE, Magee JD, et al. The RNA-Binding Protein HuR Posttranscriptionally Regulates IL-2 Homeostasis and CD4(+) Th2 Differentiation. Immunohorizons. 2017;1(6):109-23.

3. Fattahi F, Ellis JS, Sylvester M, Bahleda K, Hietanen S, Correa L, et al. HuR-Targeted Inhibition Impairs Th2 Proinflammatory Responses in Asthmatic CD4(+) T Cells. J Immunol. 2022;208(1):38-48.
