# Supplementary material for: HuR ablation destabilizes Foxp3 mRNA and impairs regulatory T cell function, contributing to an autoimmune phenotype
Source: Front Immunol. 2025 Sep 26;16:1618677. doi: 10.3389/fimmu.2025.1618677 (PMC12511036; doi:10.3389/fimmu.2025.1618677)
Supplement: Supplementary file 5 [file Table2.docx]

**Supplementary Table 2. Pathological changes of organs in Foxp3*^YFP/Cre^* HuR*^fl/fl^* mice on H&E slides (n=4 mice)**

| **Organ*** | **Pathological Observations and Severity** |
| --- | --- |
| **Bladder** | Occasional hyaline droplets in epithelium (mild) |
| **Ileum** | Segmented filamentous bacteria (SFB), mild |
| **Kidney** | Moderate-severe bilateral pyelonephritis and hydronephrosis; focal moderate unilateral pyelonephritis and hydronephrosis; occasional hyaline droplets in pelvic epithelium |
| **Liver** | Multifocal extramedullary hematopoiesis (mild-moderate); mild portal and perivascular mononuclear cell infiltrates; focal microgranuloma (mild) |
| **Lung** | Moderate-marked perivascular lymphoid infiltrates; diffuse acidophilic macrophage accumulation in alveoli; hyaline droplets in bronchiolar epithelium (mild-moderate) |
| **Lymph nodes** | Reactive |
| **Skin** | Diffuse epidermal hyperplasia with hyperkeratosis; mild-moderate increase in dermal mononuclear (primarily macrophages) and polymorphonuclear cells (dermatitis); focal edema and hyperplasia (mild overall) |
| **Spleen** | Marked extramedullary hematopoiesis; mild-moderate extramedullary hematopoiesis |
| **Stomach** | Mild focal gastritis; numerous hyaline droplets in apical enterocytes; mild patchy lymphocytic to neutrophilic infiltrates, likely incidental |

**All other examined organs, including adrenals, brain, cecum/colon, duodenum, heart, jejunum, pancreas, salivary glands, and thymus, showed no significant lesions (NSL).*
