# Supplementary material for: HuR ablation destabilizes Foxp3 mRNA and impairs regulatory T cell function, contributing to an autoimmune phenotype
Source: Front Immunol. 2025 Sep 26;16:1618677. doi: 10.3389/fimmu.2025.1618677 (PMC12511036; doi:10.3389/fimmu.2025.1618677)
Supplement: Supplementary file 6 [file Table3.docx]

**Supplementary Table 3. Molecules (n=24) associated with T helper cell differentiation pathway in YFP^+^ HuR-KO Tregs (Foxp3*^YFP/Cre^* HuR*^fl/fl^*) by IPA analysis of RNA-seq data [Ratio: 24/67 (0.358)] [z-score: NaN] [p-value: 6.91E-08].**

| □ | Symbol | Entrez Gene Name | Identifier | Expression Value | |
| --- | --- | --- | --- | --- | --- |
|  |  |  | Entrez Gene/Illumina | Expr False Discovery Rate (p-value) | Expr Fold Change |
| □ | **FCER1G*** | Fc fragment of IgE receptor Ig | **14127*** | 1.44E-02 | ↓-6.650 |
| □ | **IL18*** | interleukin 18 | **16173*** | 1.64E-02 | ↓-5.531 |
| □ | **HLA-DMB*** | major histocompatibility complex, class II, DMB | **14999*** | 2.66E-03 | ↓-3.526 |
| □ | **IL23R*** | interleukin 23 receptor | **209590*** | 4.81E-03 | ↓-3.418 |
| □ | **HLA-DQB1*** | major histocompatibility complex, class II, DQB1 | **14961*** | 7.57E-03 | ↓-3.098 |
| □ | **HLA-DQA1*** | major histocompatibility complex, class II, DQA1 | **14960*** | 4.29E-03 | ↓-3.078 |
| □ | **HLA-DRB5*** | major histocompatibility complex, class II, DRB5 | **14969*** | 1.15E-02 | ↓-2.760 |
| □ | **RORC*** | RAR related orphan receptor C | **19885*** | 1.11E-02 | ↓-2.317 |
| □ | **IL12RB2*** | interleukin 12 receptor subunit beta 2 | **16162*** | 4.66E-03 | ↓-2.236 |
| □ | **CD80*** | CD80 molecule | **12519*** | 4.71E-02 | ↓-1.876 |
| □ | **HLA-DOB*** | major histocompatibility complex, class II, DOB | **15002*** | 2.75E-03 | ↓-1.616 |
| □ | **TNFRSF1A*** | TNF receptor superfamily member 1A | **21937*** | 1.07E-02 | ↓-1.569 |
| □ | **IL10RB*** | interleukin 10 receptor subunit beta | **16155*** | 1.07E-02 | ↓-1.517 |
| □ | **ICOSLG/LOC102723996*** | inducible T-cell costimulatory ligand | **50723*** | 1.93E-02 | ↓-1.483 |
| □ | **FOXP3*** | forkhead box P3 | **20371*** | 1.65E-02 | ↓-1.374 |
| □ | **IL4R*** | interleukin 4 receptor | **16190*** | 1.29E-03 | ↓-1.344 |
| □ | **IL6ST*** | interleukin 6 signal transducer | **16195*** | 7.35E-03 | ↓-1.233 |
| □ | **TGFB1*** | transforming growth factor beta 1 | **21803*** | 4.17E-02 | ↓-1.139 |
| □ | **STAT3*** | signal transducer and activator of t | **20848*** | 1.63E-02 | ↑1.158 |
| □ | **ICOS*** | inducible T-cell costimulator | **54167*** | 3.83E-02 | ↑1.431 |
| □ | **GATA3*** | GATA binding protein 3 | **14462*** | 5.38E-03 | ↑1.473 |
| □ | **HLA-DOA*** | major histocompatibility complex, class II, DOA | **15001*** | 1.87E-02 | ↑1.622 |
| □ | **IL18R1*** | interleukin 18 receptor 1 | **16182*** | 4.69E-03 | ↑2.060 |
| □ | **IL10*** | interleukin 10 | **16153*** | 1.83E-02 | ↑2.092 |
