# Supplementary material for: HuR ablation destabilizes Foxp3 mRNA and impairs regulatory T cell function, contributing to an autoimmune phenotype
Source: Front Immunol. 2025 Sep 26;16:1618677. doi: 10.3389/fimmu.2025.1618677 (PMC12511036; doi:10.3389/fimmu.2025.1618677)
Supplement: Supplementary file 7 [file Table4.docx]

**Supplementary Table 4. Characterization of immune cell types using flow cytometry staining in various multicolor panels**

| Cell type(s) | Panel  Markers | Fluorochrome* | Vendor | Clone # | Viability Dye** |
| --- | --- | --- | --- | --- | --- |
| *CD4 T cells and CD4 Tregs* | CD3a  CD4  CD25  Foxp3 | APC-eFluor780  BV421  BV605  eFluor 660 | Invitrogen  eBioscience  BioLegend  Invitrogen | 145-2C11  RM4-5  PC61  FJK-16S | Near-IR |
| *CD8 T cells* | CD3a  CD8a | APC-eFluor780  eFluor660 | eBioscience  eBioscience | 145-2C11  53-6.7 | Violet |
| *NK and NKT cells* | CD3a  NKP46 | APC-eFluor780  eFluor 450 | Invitrogen  Invitrogen | 145-2C11  29A1.4 | Red |
| *B Cells* | CD19  B220 | AF660  APC-eFluor780 | eBioscience  eBioscience | eBio1D3  RA3-6B2 | Violet |
| *mDC and PDC* | CD11b  CD11c  Siglec H  B220 | eFluor 450  APC  PerCP/Cy5.5  APC-eFluor780 | eBioscience  Invitrogen  BioLegend  eBioscience | M1/70  N418  551  RA3-6B2 | Near-IR |
| *iMCs and Neutrophils* | CD11b  Gr-1  Ly6B.2  F4/80 | eFluor 450  AF700  PE-Vio770  APC | eBioscience  BioLegend  Miltenyi Biotec eBioscience | M1/70  RB6-8C5  REA115  BM8 | Yellow |
| *Macrophages* | CD11b  F4/80  CD68 | eFluor 450  APC  APC-eFluor780 | eBioscience  eBioscience  eBioscience | M1/70  BM8  FA-11 | Yellow |

*FITC fluorochrome was additionally applied to correspond with the YFP^+^ population in Foxp3^YFP/Cre^ HuR^fl/fl^ or the GFP^+^ population in Foxp3-GFP mice, where applicable.

**Viability Dye (LIVE/DEAD™ Fixable Stain) kit (Invitrogen, Catalog number L34960) was used, with dye selection based on panel configuration as listed above.
